# Validity of an android device for assessing mobility in people with chronic stroke and hemiparesis: a cross-sectional study

**DOI:** 10.1186/s12984-024-01346-5

**Published:** 2024-04-15

**Authors:** M. Luz Sánchez-Sánchez, Maria-Arantzazu Ruescas-Nicolau, Anna Arnal-Gómez, Marco Iosa, Sofía Pérez-Alenda, Sara Cortés-Amador

**Affiliations:** 1 Physiotherapy in Motion. Multispeciality Research Group (PTinMOTION), Department of Physiotherapy, Faculty of Physiotherapy, University of Valencia, Gascó Oliag n 5, 46010 Valencia, Spain; 2 Department of Psychology, Sapienza University of Rome, Via dei Marsi 78, 00185 Rome, Italy; 3 Smart Lab, Santa Lucia Foundation IRCCS, Via Ardeatina 306, 00179 Rome, Italy

**Keywords:** Functional mobility, Stroke, Hemiparesis, Android device, Inertial sensor, Timed up and go test, Validity

## Abstract

**Background:**

Incorporating instrument measurements into clinical assessments can improve the accuracy of results when assessing mobility related to activities of daily living. This can assist clinicians in making evidence-based decisions. In this context, kinematic measures are considered essential for the assessment of sensorimotor recovery after stroke. The aim of this study was to assess the validity of using an Android device to evaluate kinematic data during the performance of a standardized mobility test in people with chronic stroke and hemiparesis.

**Methods:**

This is a cross-sectional study including 36 individuals with chronic stroke and hemiparesis and 33 age-matched healthy subjects. A simple smartphone attached to the lumbar spine with an elastic band was used to measure participants’ kinematics during a standardized mobility test by using the inertial sensor embedded in it. This test includes postural control, walking, turning and sitting down, and standing up. Differences between stroke and non-stroke participants in the kinematic parameters obtained after data sensor processing were studied, as well as in the total execution and reaction times. Also, the relationship between the kinematic parameters and the community ambulation ability, degree of disability and functional mobility of individuals with stroke was studied.

**Results:**

Compared to controls, participants with chronic stroke showed a larger medial-lateral displacement (*p* = 0.022) in bipedal stance, a higher medial-lateral range (*p* < 0.001) and a lower cranio-caudal range (*p* = 0.024) when walking, and lower turn-to-sit power (*p* = 0.001), turn-to-sit jerk (*p* = 0.026) and sit-to-stand jerk (*p* = 0.001) when assessing turn-to-sit-to-stand. Medial-lateral range and total execution time significantly correlated with all the clinical tests (*p* < 0.005), and resulted significantly different between independent and limited community ambulation patients (*p* = 0.042 and *p* = 0.006, respectively) as well as stroke participants with significant disability or slight/moderate disability (*p* = 0.024 and *p* = 0.041, respectively).

**Conclusion:**

This study reports a valid, single, quick and easy-to-use test for assessing kinematic parameters in chronic stroke survivors by using a standardized mobility test with a smartphone. This measurement could provide valid clinical information on reaction time and kinematic parameters of postural control and gait, which can help in planning better intervention approaches.

**Supplementary Information:**

The online version contains supplementary material available at 10.1186/s12984-024-01346-5.

## Introduction

Stroke is the second-leading cause of death in adults and represents a major cause of disability worldwide [1]. There are 12.2 million new cases of stroke each year, and 101 million people are living with the consequences of a stroke [1]. This number has almost doubled in the last 30 years [1]. It is noteworthy that 70% of strokes and 87% of stroke-related deaths and disability-adjusted life-years occur in low- and middle-income countries [2]. The impact of persistent stroke-related disability is significant to both individuals and society. Therefore, there is an ongoing need to address the challenges faced by stroke survivors [3]. Stroke typically causes sensorimotor deficits such as muscle weakness in the paretic limbs, impaired proprioceptive capabilities, sensory loss, vision problems, and spasticity [4]. These deficits affect the postural stability of stroke survivors and have significant influence on their mobility [5], causing functional mobility impairment after stroke [6].

Since functionality is related to daily life activities (i.e., sitting and standing, walking, turning, and going up and down stairs) [6], its impairment negatively affects the quality of life of post-stroke subjects [7]. Furthermore, functional mobility in the community requires not only the ability to maintain balance while walking and turning, but also to ambulate at 0.80 m/s or faster [8, 9]. Thus, difficulties with mobility control, particularly in balance and gait, are a priority clinical concern in this population [10].

In that sense, monitoring the improvement of functional mobility post-stroke is relevant as it could help to determine the effect of rehabilitation and thus expand therapeutic options [11–13]. The gold standard measurement of functional mobility is photogrammetry [14]. However, it is time-consuming and difficult to perform in clinical practice. Therefore, several tests have been used in clinical settings and research to determine functional mobility in stroke. Particularly, the Time Up and Go Test (TUG) [7], the Tinetti’s Scale of Mobility and Balance [7], the Dynamic Gait Index [15] and the Rivermead Mobility Index [16, 17]. These tools are quick and easy to use and do not require expensive materials. However, they provide a subjective evaluation and are less sensitive in detecting changes [18]. Therefore, incorporating instrumental systems into clinical assessments could improve the accuracy of results. These devices provide objective information on biomechanical aspects such as posture, movement, and compensatory strategies [18].

In this regard, Lin et al. [5] compared the results of clinical assessment scales (lower extremity subscale of the Fugl-Meyer Assessment, Berg Balance Scale and TUG) versus instrumental balance assessment (stability, proprioception and limits of stability) and showed that the later was superior to clinical alternatives in detecting balance impairments in stroke patients with mild balance disorders. As a conclusion, these authors recommended that clinicians consider the use of both classic clinical tests and quantitative biomechanical tools when evaluating stroke patients to improve the accuracy of assessments, leading to a better individualized treatment plan [5]. Similarly, the Stroke Recovery and Rehabilitation Roundtable of the International Stroke Recovery and Rehabilitation Alliance considered kinematic measures to be essential in assessing sensorimotor recovery [19]. Therefore, the use of a device that can help measure kinematic data when performing several daily activity tasks (such as rising, walking, turning, and sitting down) in a single test could help clinicians in evidence-based decision making. For such a purpose, current evidence examines various technological approaches. Many of them are not portable or easy to move, and the evaluation procedure is complex and time-consuming [20]. As a result, researchers have begun using smartphone applications to assess walking activity and balance performance in stroke [21].

Smartphones are equipped with advanced computing capabilities, global positioning system receivers, and sensing capabilities such as an inertial measurement unit (IMU), magnetometer, and barometer [22]. These features can also be found in wearable ambulatory monitors [22]. Hence, smartphones are currently gaining interest in post-stroke clinical assessment due to their validity, reliability, portability and price [21, 23], not only for measuring upper limb range of motion [24] but particularly for gait and balance assessment [21]. A systematic review of smartphone technology [25] found that a few systems have utilized IMU sensors embedded in smartphones, while more systems have integrated external sensors for data acquisition and used the smartphone as a data processing unit in stroke patients. For gait and balance assessment, smartphones has primarily been used to determine step counts and cadence [26, 27], recognize human movement activity [28], study posturography [20, 29], and analyze kinematic data during gait [23, 30, 31]. It should be noted that the current scientific evidence indicates that more studies are needed [20, 21], especially with standardized protocols and appropriate sample calculations [21]. To the best of our knowledge, no study has been found that uses a smartphone in a single test to evaluate kinematic data during the joint performance of different tasks relevant to activities of daily living, such as reaction time, sitting down, getting up or turning around, in stroke population. Therefore, this study aimed to assess the validity of using an Android device to evaluate kinematic data during the execution of a standardized mobility test that includes various tasks relevant to activities of daily living in people with chronic stroke and hemiparesis. To achieve this objective, the data registered by the sensor were compared between stroke and non-stroke individuals. In addition, the relationship between clinical tests and kinematic parameters were studied for patients. If our working hypothesis is met, the inclusion of an inertial sensor on an Android device when performing a standardized mobility test will provide further information about which daily activity tasks have greater impairment in the population with chronic stroke, without the need to allocate more time for the clinical assessment.

## Materials and methods

### Study design and participants

This is a cross-sectional design study that included individuals with stroke in chronic phase (onset > 6 months) and hemiparesis as well as age-matched healthy subjects without a history of falls in the preceding year. Adults with stroke were recruited from several brain injury associations of patients located in the region of Valencia (Spain), and the healthy participants were recruited from the authors’ institution and through personal contact. Recruitment was performed from January 2022 to March 2023. Inclusions were made if participants had motor or sensorimotor hemiparesis affecting only the lower limb or both the lower and upper limbs, could walk for at least 10 m with or without an assistive device or supervision (functional ambulation classification of the Hospital of Sagunto [FACHS] ≥ 2 [32]), did not present with severe disability (modified Rankin scale [mRS] ≤ 3 [33]), and had the ability to understand verbal instructions to undergo the assessment tests (evaluated by a neuropsychologist expert in brain injury). Exclusions were made in cases of vestibular, neurological (e.g. Parkinson’s disease, ataxia), or severe musculoskeletal conditions (e.g. recent surgery, amputations) and pain (visual analogue scale ≥ 3 [34]) that interfere with mobility, as well as any other impairment that precluded participants from performing the assessment tests.

Prior sample size was estimated with the GRANMO® calculator (*Institut Municipal d’Investigació Mèdica*, Barcelona, Spain, Version 7.12). For this purpose, data of a previous pilot study was used [35]. Specifically, the sample size was calculated based on the mean difference between stroke and non-stroke subjects in the time required to perform the standardized mobility test. Setting both alpha and beta errors at 0.05 in a two-sided test, assuming a common deviation of 10.11 s [35] to be recognized as statistically significant, and ascertaining a between-group difference of ≥ 9.57s [35], established that 30 subjects per group were needed (60 total).

All study participants were fully informed about the purpose of the study and the experimental procedure, and provided written informed consent. The study conformed to the Declaration of Helsinki and was approved by the Ethics Committee of Human Research (H1417615024926) of the authors´ institution. This article adheres to the STROBE guidelines [36].

### Experimental procedures

A single-session assessment was arranged for each participant. They were asked to attend the session in comfortable clothing and well-fitting footwear, and to avoid vigorous physical activity on the assessment day. Ankle foot orthoses were allowed if necessary.

Sociodemographic and clinical data were collected from a clinical interview and medical records. Muscle spasticity of the paretic calf was measured with the modified Ashworth scale (MAS) [37], the ability to walk was assessed with the functional ambulation classification of the Hospital of Sagunto (FACHS) [32], disability was evaluated with the mRS [33] and cognitive status was assessed with the Montreal Cognitive Assessment [38]. Height and weight were measured and body mass index (BMI) was calculated. Then, participants went on to perform the assessment tests.

### Functional mobility tests

According to the functional tasks involved in the standardized mobility test, three tests were performed:

1. *The Timed Up and Go test (TUG)* [39]. This is a frequently used test in clinical settings that addresses basic mobility skills. Participants were asked to stand up from a chair, walk a 3-meter distance at a comfortable and safe walking speed, turn, walk back and sit down on the chair. Participants were timed from when they stood up to when they sat down, and this time was recorded. A trial test was allowed before timing the test and for familiarization. A lower time is indicative of a better outcome. The TUG has shown excellent intra-rater (intraclass correlation coefficient [ICC]: 0.95–0.96) and inter-rater (ICC: 0.97–0.99) reliabilities in stroke survivors [40].
2. *The 10-meter walk test* (10MWT) [41]. This test was employed to measure walking speed. Participants were asked to walk at a comfortable peace along a 10-meter walkway. The time taken to walk the 6 central meters was measured, as 2-meter distances at the beginning and at the end of the walkway were allowed for acceleration and deceleration, respectively. The test was performed twice, and the shortest time was registered. The walking speed was then calculated and used for statistical purposes; a higher walking speed indicated a better ambulation capacity. This test has been found to have excellent test-retest (ICC: 0.85–0.97), intra-rater (ICC: 0.92–0.94), and inter-rater (ICC: 0.96–0.97) reliabilities in people with chronic stroke [41].
3. *The five times sit-to-stand test* (5xSTS) [42]. This test was used to measure sit-to-stand ability. Starting at a sitting position with the back against the back of a standard-height chair, participants were instructed to stand up and sit down five times as quickly and safely as possible without using the arms for support. The amount of time required to perform the five repetitions was measured and registered, with lower times indicating better outcomes. Reliability of the 5xSTS has been found to be excellent (ICCs for intra-rater, inter-rater, and test-retest ranging from 0.970 to 0.999) in populations with chronic stroke [43].

### Standardized mobility test

The standardized mobility test was conducted using an inertial sensor (High Performance 6-Axis MEMS MotionTracking™ composed of a 3-axis gyroscope, 3-axis accelerometer and a Digital Motion Processor™ [TDK- ICM-20689) at 100 Hz] embedded in an Android device smartphone (Xiaomi Redmi 4x Model MAG138). Only one type of smartphone was used in this study because literature reports only small differences among different models: about 0.3 m/s in velocity, one degree in roll and pitch [44] and 0.3 degrees in the Root Mean Square mean values for static protocols [45]. For dynamic conditions, differences may increase and depend on the sampling frequency, the acceleration sensitivity, and especially of the data analysis protocol [46]. These differences are similar to those also found among various types of commercial IMUs, whether integrated into smartphones or not [47]. Throughout the test, the inner sensor registered the accelerations generated by each participant´s movement while the sensor signals were recorded with the FallSkip® app system (*Instituto de Biomecánica de Valencia*, Valencia, Spain). The system’ s raw data (recordings of measurements and testing times) were used in this study and were processed on a custom specific software (see sensor data processing section). The Fallskip® measurement system was validated against the Kinescan/IBV v7.0 photogrammetry system (*Instituto de Biomecánica de Valencia*, Valencia, Spain), and all parameters showed ICCs greater than 0.7 [48].

**Fig. 1:**
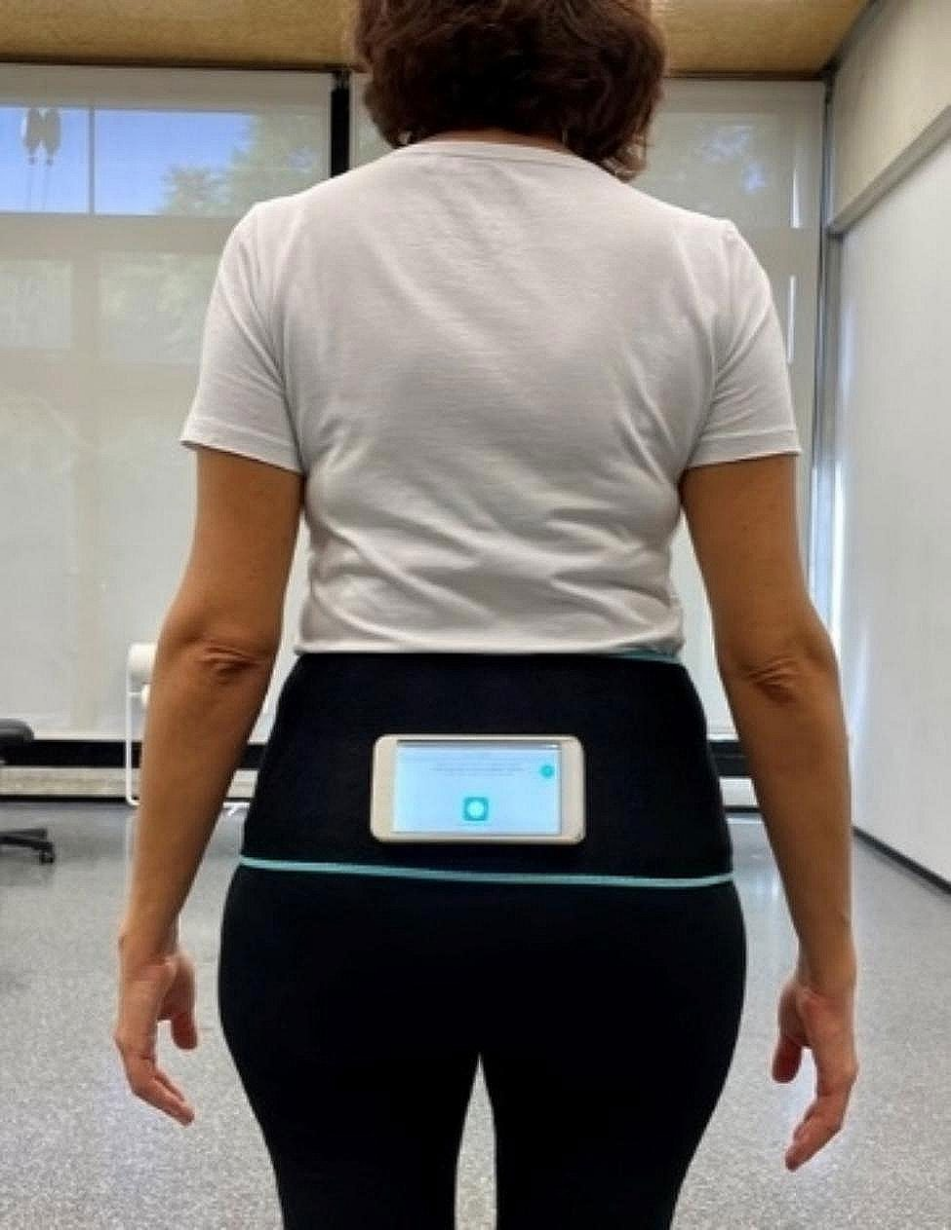
Position of the Android smartphone

To perform the standardized mobility test, the protocol developed for the FallSkip® system was used [49]. In the assessment procedure, the test was first explained and demonstrated. Next, participants were equipped with a lumbar belt with the Android device fixed horizontally and parallel to the ground so that the upper edge was aligned with the joining of the posterior-superior iliac spines (Fig. 1). The positioning and fixation of the device in correspondence with the center of mass (COM) of the whole body, by means of an elastic belt able to secure it and to avoid any displacement, was in line with previous literature on the instrumental assessment of walking ability and stability in patients with stroke performed with a single wearable device [50–57]. The validity of this approach with specially developed wearable devices fixed with an elastic belt had already been demonstrated, but the use of a smartphone, which incorporates an inertial unit and could allow for a more widespread use of this technique, had not been tested.

**Fig. 2:**
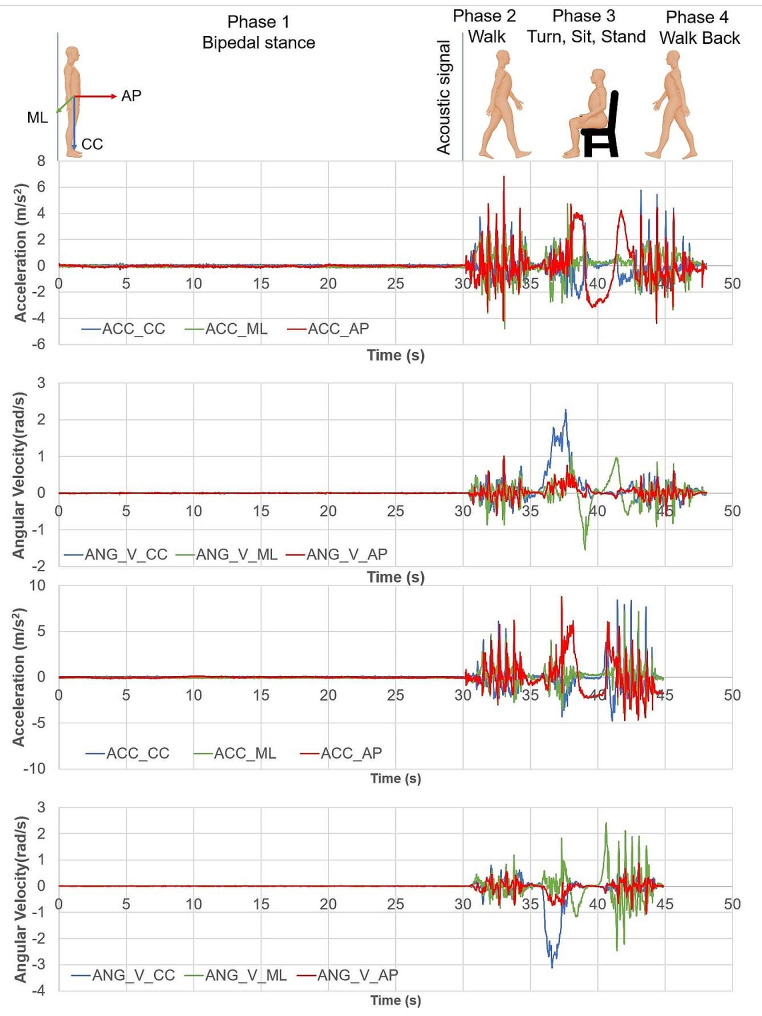
Graphical presentation of the sensor data recordings during each phase of the mobility test. Data of a 65-year old male participant with stroke is displayed in the upper two graphs and data of his healthy age-matched control is displayed in the bottom two graphs. ML: Medial-lateral axis; AP: Anterior-posterior axis; CC: Cranio-caudal axis; ACC: acceleration; ANG_V: angular velocity

Then, the four phases of the test were consecutively performed in a single recording (Fig. 2):

- Phase 1: Bipedal stance. The measurement started with the participant standing still with arms hanging relaxed at the sides of the body for 30 s.
- Phase 2: Walking. At the sound of an acoustic signal, the participant was required to immediately start walking and go through a 3-meter corridor straight toward a chair.
- Phase 3: Sitting down on a chair and standing up from it. At the end of the corridor, the participant had to stop for three seconds, turn around, sit down on the chair, remain seated for three seconds and then stand up from the chair.
- Phase 4: Walking. Finally, the participant had to walk back to the starting point.

A single evaluator performed all the tests. During the whole test, the evaluator walked one meter away from the participant as a precautionary measure. In elderly people, this assessment protocol has shown good to excellent test–retest reliability for all the kinematic variables (ICC: 0.75–0.93) [58].

### Sensor data processing

**Fig. 3:**
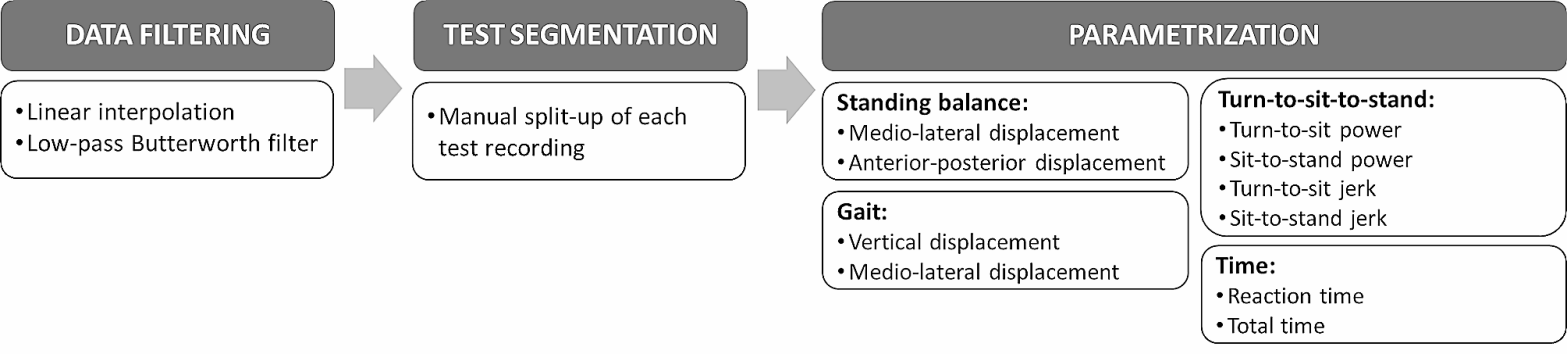
Sensor data processing procedure

All the raw data acquired by the sensor were processed on custom specific offline Python (3.x) scripts according to the procedure showed in Fig. 3.

### Data filtering

To filter the raw sensors signals, the study utilized the methodology for inertial sensor data analysis proposed by Pedrero-Sánchez et al. [59], which built on Zijlstra´s [60] and Nishiguchi et al.´s [61] work. The data registered by the Android device inertial sensor were collected at a fixed sampling rate of 100 Hz. To assure that data points were equally distributed in time and that information was not missed between consecutive samples, an interpolation of the registered signal was performed. Then, signals were filtered with a low-pass Butterworth filter (fourth-order zero-lag at 20 Hz).

### Test segmentation

To identify the time events related to the tasks under interest, each test recording was manually split up by the same individual. To this end, the beginning and ending of each phase was determined by using a graph plotting the data vectors of accelerometers, gyroscopes and magnetometer. The segmentation process utilized the following key moments of the test [58, 62]:

t0, conclusion of the postural control test 30 s after initiation.

t1, onset of walking identified by the increase in activity of acceleration (acc) signals.

t2, cessation of walking indicated by the stabilization of acc signals near baseline (t0) values, preceding a 3-second pause.

t3, beginning of turning displayed by a constant change in acc and/or gyroscope (gyro) magnitudes.

t4, completion of the sitting down phase identified by the stabilization of both acc and gyro signals, preceding the 3-second pause.

t5, initiation of standing up displayed by a consistent change in both acc and gyro magnitudes.

t6, conclusion of the standing up phase characterized by the return to baseline (t0) values of both acc and gyro magnitudes.

t7, beginning of walking backward, determined by either at t6 or when acc signals increase after the pause.

t8, termination of the backward walking phase, characterized by the stabilization of acc signals close to baseline (t0) values.

### Parametrization

In this phase, kinematic parameters (per phase and for the complete test) were computed based on previously validated research [60, 61, 63, 64]. The sensor’s orientation was determined from the accelerations and angular velocities using Favre’s method [65]. The orientations were expressed in Euler angles (Roll, Pitch, and Yaw) and quaternions (qw, qx, qy, and qz). Orientation was used to segment the test phases and identify the moments of directional change, such as turning to sit. The sensor’s position was then calculated analytically in the frequency domain by double integrating the acc signal using the Fourier transform and its inverse, as described by Ribeiro et al. [63]. The purpose of this approach was to minimize the signal shift when double integrating acc signals to determine displacement. Hence, kinematic variables were extracted from the various position signals in the different phases of the test. Table 1 shows the dependent variables and their respective methods of acquisition (please, refer to supplementary material 1 for further detail of calculating the kinematic variables).

**Table 1:** Kinematic parameters calculated

Variable	Description	Calculation	Measurement units
**Assessment of postural control**			
Medial-lateral displacement (MLDisp)	Medial-lateral excursion of the COM during the 30 s bipedal phase.	90th percentile of the double integration of the acc signal [63] and an inverted pendulum model [60]	mm
Anterior-posterior displacement (APDisp)	Anterior-posterior excursion of the COM during the 30 s bipedal phase.		mm
**Assessment of gait**			
Cranio-caudal range (CCrange)	Vertical COM movement, taking the average of walking forth (t1,t2)* and back (t7,t8)* over the 3-meter distance.	90th percentile of the double integration of the acc signal [64]	mm
Medial-lateral range (MLrange)	Horizontal COM movement, taking the average of walking forth (t1,t2)* and back (t7,t8)* over the 3-meter distance.		mm
**Assessment of turn-to-sit-to-stand**			
Turn-to-sit power (PturnSit)	Mean power generated when turning around and sitting on the chair (t3,t4)*.	Estimated by the trajectory of the COM during movement, participant’s weight and height, and the time taken for stand-to-sit and sit-to-stand [66].	Watts
Sit-to-stand power (Pstand)	Mean power generated by getting up from the chair (t5,t6)*.		Watts
Turn-to-sit jerk (RangeJerkSit)	Range of over acceleration when turning around and sitting on the chair (t3,t4)*.	Obtained by subtracting the maximum minus the minimum of the Jerk during the gesture.	m/s^3^
Sit-to-stand jerk (RangeJerkStand)	Range of over acceleration when getting up from the chair (t5,t6)*.		m/s^3^
**Assessment of time**			
Total execution time	Time needed to complete all the phases of the test.	Sum of the split of walking (t1,t2 and t7,t8)* and sit (t3,t4)* to stand times (t5,t6)*.	seconds
Reaction time	Time elapsed from the acoustic signal to gait initiation (t0,t1)*.		seconds

* timing used during the segmentation process (please, refer to the test segmentation section)

COM: Center of mass; acc: acceleration

The variables selected for this study and used for statistical analysis were based on scientific literature. These variables include the displacements along body axes of the COM during posture and gait [67] and the time for executing the turn-to-sit-to-stand [68], its relative power, and the jerk that is the derivate in time of acceleration [69]. Medial-lateral and anterior-posterior displacements are commonly measured to reflect steadiness in terms of COM displacement during postural control assessment [70]. The cranio-caudal range can be considered a measure of energy cost [64, 71] and may relate to walking efficiency [62]. Additionally, the medial-lateral range, along with energy cost, represents the ability to control body movement during gait [72]. The turn-to-sit task and the sit-to-stand task necessitate cognitive planning and a proper neuromuscular system coordination to control COM displacement and postural alignment [48]. Finally, reaction time is indicative of the cognitive processing required for postural control after a stroke [73].

### Statistical analyses

For the descriptive analysis, continuous data are reported as mean (standard deviation) or median (interquartile range) while categorical data are shown as percentage. The normality of continuous data was checked with the Kolmogorov-Smirnov test and the Shapiro-Wilk test, as appropriate.

To assess validity, the differences in the dependent variables obtained after sensor data processing (Table 1) between the stroke group versus the non-stroke group were first studied by using a series of independent t-tests or Mann-Whitney U tests, as appropriate. Effect size statistics (r) were also calculated and interpreted as *r* = 0.12 small effect, *r* = 0.20 medium effect, and *r* ≥ 0.32 large effect [74]. Second, the association between the functional mobility tests (TUG time, 10MWT walking speed and 5xSTS time) and the kinematic variables (Table 1) was established by using the Pearson´s or Spearman’s Rho correlation coefficient (ρ), interpreting 0.1 < ρ < 0.3 as small, 0.4 < ρ < 0.6 as medium and ρ ≥ 0.7 as large [75]. Although multiple correlation tests were performed, they did not refer to a single specific null hypothesis, so no adjustments were adopted to the alpha level of significance [76]. Third, for disability (mRS) and ambulation ability (FACHS), stroke participants were divided into two groups: those without significant disability (mRS = 0–1) or slight/moderate disability (mRS = 2–3) for the former; and those with independent community ambulation (FACHS = 4 or 5 points) or limited community ambulation (FACHS = 2 or 3 points) for the latter. Then, independent t-tests or Mann-Whitney U tests were used to compare the kinematic variables between groups, and the effect size statistics (r) were calculated.

Statistical analyses were performed with IBM SPSS Statistics software (SPSS Inc., Chicago, IL, USA, v.28). Significant results were reported if *p* < 0.05.

## Results

### Participants

Thirty-six individuals with chronic stroke (mean age 58.5 ± 8.7 years; 25% women) and 33 healthy counterparts (mean age 61.1 ± 10.1 years; 45.5% women) were enrolled in the study. Table 2 shows the participants’ characteristics. There were significant differences between groups in BMI (*p* = 0.001), cognitive status (*p* < 0.001), degree of disability (*p* < 0.001) and ambulation ability (*p* < 0.001), with better results in the non-stroke group. Functional mobility tests were significantly different between groups (*p* < 0.001), with the stroke group showing worst results. In the stroke group, the average time since stroke was 68.8 ± 47.5 months, 58.33% of participants had left hemiparesis and 83.4% had suffered a single stroke.

**Table 2:** Characteristics of the participants

	Stroke group (*n* = 36)		Non-stroke group (*n* = 33)		Between-group differences		
					t/U/χ^2^	P	ES
**Demographics and anthropometrics**							
Age, *mn ± SD*	58.5 ± 8.7		61.1 ± 10.1		1.148	0.258	0.139
Men/women, *%*	75/25		54.5/45.5		3.176	0.075	
BMI, *mn ± SD*	29.01 ± 5.53		25.17 ± 3.71		3.353	**0.001**	0.377
**Clinical characteristics**							
Cognitive status – MOCA, *mn ± SD*	22.81 ± 3.53		26.21 ± 2.60		4.534	**< 0.001**	0.485
Disability-mRS, *md (IQR)*	2 (1–2)		0 (0–0)		49.500	**< 0.001**	
Ambulation ability*- FACHS, md (IQR)*	4 (3–4)		5 (5–5)		115.500	**< 0.001**	
TUG Time (s)	11.6 (9.6–14.7)		7.7 (6.7–8.3)		141	**< 0.001**	0.655
Walking speed- *10MWT* (m/s), *mn ± SD*	0.95 ± 0.32		1.37 ± 0.15		7.012	**< 0.001**	0.257
Sit-to-stand ability − *5xSTS* (s), *md (IQR)*	13.98 (12.53–18.29)		11.28 (9.46–12.84)		212.5	**< 0.001**	0.551
**Stroke Characteristics**							
More than 1 stroke, *%*	16.6						
Left hemiparesis, *%*	58.33						
Poststroke duration (in months), *mn ± SD*	68.8 ± 47.5						
Muscle spasticity paretic calf- *MAS*, *%*							
0	41.67						
1	11.11						
1+	27.78						
2	11.11						
3	5.56						
4	2.78						

Data are expressed as mean (mn) ± standard deviation (SD), median (md) (interquartil range [IQR]) or otherwise stated. Significant differences are highlighted in bold. Between-group differences were calculated by using the independent t-test or the Mann-Whitney U test for continuous data and the Chi Squared test, for categorical data. ES: r effect size statistics (*r* = 0.12 small effect; *r* = 0.20 medium effect; *r* ≥ 0.32 large effect [74]). BMI: Body mass index; MOCA: Montreal Cognitive assessment; mRS: modified Rankin scale; FACHS: functional ambulation classification of the Hospital of Sagunto; TUG: Timed up and go test; 10MWT: 10-meter walk test; 5xSTS: five times sit-to-stand test; MAS: modified Ashworth scale

### Validity results

The inferential analysis comparing the dependent variables between groups (stroke versus non-stroke) is displayed in Table 3. There was statistical significance in the between-group differences in the medial-lateral displacement (postural control assessment) (*p* = 0.022), the cranio-caudal and medial-lateral ranges (gait assessment) (*p* = 0.024 and *p* < 0.001, respectively), in the turn-to-sit power (*p* = 0.001), turn-to-sit jerk (*p* = 0.026) and sit-to-stand jerk (*p* = 0.001) (assessment of turn-to-sit-to-stand), and in the total execution and reaction times (*p* < 0.001 in both cases). Compared to the non-stroke group, the stroke participants showed a greater medial-lateral displacement and medial-lateral range, shorter cranio-caudal range, lesser turn-to-sit power, turn-to-sit jerk and sit-to-stand jerk, and an increased total time and reaction time.

**Table 3:** Between-group differences in the kinematic variables

	Stroke group (*n* = 36)		Non-stroke group (*n* = 33)		Between-group differences		
					t_(67)_/U	P	ES
**Android device: assessment of postural control**							
Medial-lateral displacement (mm)	6.76 (4.71–9.65)		5.17 (3.04–7.82)		404	**0.022**	0.275
Anterior-posterior displacement (mm)	14.34 (11.89–19.63)		13.53 (10.43–20.57)		549	0.589	0.065
**Android device: assessment of gait**							
Cranio-caudal range (mm)	29.08 (23.05–40.62)		38.13 (28.71–50.65)		406	**0.024**	0.272
Medial-lateral range (mm)	74.29 (60.56–99.82)		56.22 (38.93–63.61)		304	**< 0.001**	0.419
**Android device: assessment of turn-to-sit-to-stand**							
Turn-to-sit power (W)	100.77 ± 37.08		137.54 ± 46.52		3.646	**0.001**	0.407
Sit-to-stand power (W)	224.39 ± 95.04		250.74 ± 84.58		1.212	0.23	0.147
Turn-to-sit jerk (m/s^3^)	18.42 (15.04–23.91)		23.31 (17.06–32.46)		409	**0.026**	0.268
Sit-to-stand jerk (m/s^3^)	24.12 ± 9.05		31.64 ± 9.13		3.434	**0.001**	0.387
**Android device: assessment of time**							
Total time (s)	15.54 (12.81–17.52)		10.21 (9.71–11.13)		104.5	**< 0.001**	0.708
Reaction time (s)	1.15 ± 0.48		0.77 ± 0.30		3.981	**< 0.001**	0.437

Data are expressed as mean (mn) ± standard deviation (SD) or median (md) (interquartil range [IQR]). Significant results are highlighted in bold. Between-group differences were calculated by using the independent t-test (t) or the Mann-Whitney U test (U).

ES: r effect size statistics (*r* = 0.12 small effect; *r* = 0.20 medium effect; *r* ≥ 0.32 large effect [74])

When the Android device’s performance was evaluated against the TUG in the stroke group (Table 4), the TUG time correlated positively and significantly with the medial-lateral range (*p* < 0.001) and the total execution time (*p* < 0.001). A negative and significant correlation was found between the TUG time and the turn-to-sit power (*p* = 0.031), the sit-to-stand power (*p* = 0.038), and the sit-to-stand jerk (*p* = 0.039).

**Table 4:** Correlation results between the functional mobility tests and the kinematic variables in the stroke group (*n* = 36)

		MLDisp	APDisp	CCrange	MLrange	PTurnSit	PStand	Range JerkSit	Range JerkStand	Total time	Reaction time
TUG time	Correlation coefficient	− 0.070	0.082	0.077	,661	-,360	-,347	− 0.296	-,345	,847	0.236
	p	0.684	0.635	0.657	**< 0.001**	**0.031**	**0.038**	0.080	**0.039**	**< 0.001**	0.165
Walking speed (10MWT)	Correlation coefficient	0.000	− 0.085	0.013	− 0.569	.287^a^	.278^a^	0.103	.323^a^	− 0.752	− .229^a^
	p	0.999	0.622	0.942	**< 0.001**	0.089	0.101	0.550	0.055	**< 0.001**	0.179
5xSTS time	Correlation coefficient	− 0.117	0.346	− 0.278	0.367	− 0.283	− 0.162	− 0.435	− 0.411	0.577	0.502
	p	0.497	**0.039**	0.101	**0.028**	0.095	0.344	**0.008**	**0.013**	**< 0.001**	**0.002**

^a^ Association assessed with the Pearson´s correlation coefficient.

TUG: Timed up and go test; 10MWT: 10-meter walk test; 5XSTS: five times sit-to-stand test; MLDisp: Medial-lateral displacement; APDisp: Anterior-posterior displacement; CCrange: Cranio-caudal range; MLrange: Medial-lateral range; PTurnSit: Turn-to-sit power; PStand: Sit-to-stand power; RangeJerkSit: Turn-to-sit jerk; RangeJerkStand: Sit-to-stand jerk.

Significant results are highlighted in bold.

Table 4 also illustrates the results of the correlation analyses between the kinematic variables and both the walking speed (10MWT) and the sit-to-stand ability (5xSTS) in the stroke group. Significant negative correlations were found between walking speed and the medial-lateral range (*p* < 0.001) and total execution time (*p* < 0.001). The 5xSTS time correlated significantly and positively with the anterior-posterior displacement (*p* = 0.039), the medial-lateral range (*p* = 0.028) and the total execution and reaction times (*p* < 0.001 and *p* = 0.002, respectively). It correlated significantly and negatively with the turn-to-sit jerk (*p* = 0.008) and sit-to-stand jerk (*p* = 0.013).

When participants with stroke were grouped according to their degree of disability (mRS; Table 5), significant between-group differences were found for the medial-lateral range (*p* = 0.024), total execution time (*p* = 0.041) and reaction time (*p* = 0.040). These three variables showed higher values in stroke participants with slight/moderate disability.

**Table 5:** Differences in the kinematic variables according to the degree of disability (mRS) of stroke participants (*n* = 36)

	Stroke group without significant disability (mRS = 0–1) (*n* = 11)		Stroke group with slight/moderate disability (mRS = 2–3) (*n* = 25)		Between-group differences		
					t_(34)_/U	P	ES
**Android device: assessment of postural control**							
Medial-lateral displacement (mm)	5.25 (3.51–8.48)		7.18 (5.01-10)		101	0.210	0.214
Anterior-posterior displacement (mm)	13.27 (10.97–17.95)		16.51 (12.13–24.12)		87	0.083	0.297
**Android device: assessment of gait**							
Cranio-caudal range (mm)	31.73 (22.93–42.07)		27.56 (23-37.43)		118	0.503	0.114
Medial-lateral range (mm)	61.38 (51.44–75.15)		77.78 (65.15-104.96)		72	**0.024**	0.385
**Android device: assessment of turn-to-sit-to-stand**							
Turn-to-sit power (W)	103.84 ± 30.72		99.41 ± 40.07		0.326	0.747	0.055
Sit-to-stand power (W)	216.15 (167.31-278.69)		191.34()		129	0.770	0.050
Turn-to-sit jerk (m/s^3^)	19.38 (16.43–25.36)		18.30 ()		108	0.311	0.173
Sit-to-stand jerk (m/s^3^)	27.53 ± 11.31		22.61 ± 7.65		1.530	0.135	0.253
**Android device: assessment of time**							
Total time (s)	12.89 (10.36–16.34)		16.26 (14.20-19.46)		78	**0.041**	0.350
Reaction time (s)	0.91 ± 0.27		1.26 ± 0.51		2.133	**0.040**	0.343

Data are expressed as mean (mn) ± standard deviation (SD) or median (md) (interquartil range [IQR]). mRS: modified Rankin Scale Between-group differences were calculated by using the independent t-test (t) or the Mann-Whitney U test (U). Significant results are highlighted in bold. ES: r effect size statistics (*r* = 0.12 small effect; *r* = 0.20 medium effect; *r* ≥ 0.32 large effect [74]).

Regarding the comparison of stroke participants according to their community ambulation ability (FACHS; Table 6), there were statistically significant differences for the medial-lateral range (*p* = 0.042) and the total time (*p* = 0.006) between groups, with stroke participants with limited community ambulation showing higher values in both variables.

**Table 6:** Differences in the kinematic variables according to the community ambulation ability (FACHS) of stroke participants (*n* = 36)

	Stroke group with independent community ambulation (*n* = 21)		Stroke group with limited community ambulation (*n* = 15)		Between-group differences		
					t_(34)_/U	P	ES
**Android device: assessment of postural control**							
Medial-lateral displacement (mm)	6.59 (4.33-13,76)		6.92 (4.74–9.59)		153	0.885	0.024
Anterior-posterior displacement (mm)	13.89 (11.30-19.38)		14.47 (13.46–22.45)		135	0.470	0.123
**Android device: assessment of gait**							
Cranio-caudal range (mm)	28.55 (23.17–36.46)		33.11 (22.43–41.94)		130	0.378	0.151
Medial-lateral range (mm)	66.57 (52.21–94.71)		78.31 (67-112.71)		94	**0.042**	0.349
**Android device: assessment of turn-to-sit-to-stand**							
Turn-to-sit power (W)	102.46 ± 30.59		98.39 ± 45.72		0.320	0.751	0.054
Sit-to-stand power (W)	216.15 (169.54-278.74)		179.88 (132.85-247.95)		118	0.205	0.217
Turn-to-sit jerk (m/s^3^)	21.03 ± 6.91		18.28 ± 7.19		1.156	0.256	0.194
Sit-to-stand jerk (m/s^3^)	25.96 ± 10.05		21.54 ± 6.93		1.468	0.151	0.244
**Android device: assessment of time**							
Total time (s)	14.14 ± 2.71		18.91 ± 5.57		3.063	**0.006**	0.577
Reaction time (s)	1.09 ± 0.38		1.24 ± 0.60		0.968	0.340	0.163

Data are expressed as mean (mn) ± standard deviation (SD) or median (md) (interquartil range [IQR]). Between-group differences were calculated by using the independent t-test (t) or the Mann-Whitney U test (U). Significant results are highlighted in bold. ES: r effect size statistics (*r* = 0.12 small effect; *r* = 0.20 medium effect; *r* ≥ 0.32 large effect [74])

## Discussion

Based on our results, a standardized mobility test performed with a smartphone inertial sensor is a valid, single, quick and easy-to-use test that assesses kinematic parameters in various tasks related to activities of daily living in people with chronic stroke. Compared to their age-matched healthy counterparts, people with chronic stroke showed motor impairment in gait, turning and sitting, reaction time, and total execution time. In addition, a relationship between some of these parameters was demonstrated with physical functions usually related to impaired mobility after stroke. Likewise, the medial-lateral range of the COM when walking was found to be a relevant parameter for distinguishing between independent and limited community ambulation patients and stroke participants with significant disability or slight/moderate disability.

Consistent with previous evidence [77], stroke participants in our study took more time than healthy controls when performing both the standardized mobility test with the Android device and the functional mobility tests (TUG, 10MWT, and 5xSTS). Besides the increased time to perform the tests, the Android device showed that participants with hemiparesis had greater medial-lateral displacement of their COM while standing and walking. They also showed lower cranio-caudal COM movement when walking, lower power and range of over acceleration when turning and sitting, and lower range of over acceleration when getting up.

In the stroke group, some statistically significant relationships were found between the clinical tests (TUG, 10MWT, and 5xSTS) and some of the kinematic parameters reported by the Android device (mainly, medial-lateral range when walking and total execution time). These relationships indicated that the more clinical impairment a subject showed, the more different these kinematic parameters would be. Finally, the medial-lateral range of COM during walking and the total execution time of the test seem to be the variables that discriminate the most between stroke participants with independent community ambulation (FACHS = 4 or 5 points) and those with limited community ambulation (FACHS = 2 or 3 points). Similarly, these two variables and the reaction time discriminated between stroke participants without significant disability (mRS = 0–1) and those with slight/moderate disability (mRS = 2–3). These study’s results are significant because a recent systematic review on smartphone-based gait and balance assessment in stroke survivors emphasized the importance of evaluating smartphone applications to differentiate between varying levels of impairment [21].

Postural stability when standing has been frequently studied post-stroke using force platforms to measure the center of pressure (COP) trajectories [78]. In line with previous studies that have determined greater displacements of the COP in people with chronic stroke [79, 80], our participating chronic stroke survivors also displayed higher values of COM displacement in the medial-lateral direction. This is an interesting result since balance recovery post-stroke is characterized by a reduction in postural sway and instability, particularly in the frontal balance plane [81]. In this regard, it should be noted that although during posturography subjects typically maintain their feet in a standardized position, the methodology of our test considers comfortable foot positioning during the bipedal stance. A recent study [80] determined that, in the stroke population, standardization of position does not lead to reduced variability in the test. Thus, adoption of a comfortable position might be beneficial since it allows for a more practical and realistic evaluation of postural control.

Chronic stroke survivors exhibited altered gait, as was indicated by a higher medial-lateral range and a lower cranio-caudal range of COM when walking [82]. The cranio-caudal range could be considered a measure of energy cost [64, 71], while the medial-lateral range, in addition to energy cost, is representative of the dynamic stability during walking. In that case, the higher medial-lateral range demonstrated by the stroke participants could be explained because, to maintain stability during locomotion, effective neuromotor control of the lower extremities contributes to regulating the COM position and movement relative to the base of support. Thus, compared to healthy controls, chronic stroke subjects have a reduced capacity to rapidly shift their COP to the stance limb during gait initiation, which reflects abnormalities in balance control during weight transfer [83]. Walking and other common daily activities require constant COP shifting within the limits of body stability in both the anteroposterior and mediolateral directions. Subjects with greater COP displacement are more unstable [84]. Other authors reported alterations in the COM trajectory of post-stroke subjects when walking in order to identify postural control impairments [85, 86]. However, such researchers use photogrammetry during gait studies in a laboratory setting. The novelty of our study is that it considers the analysis of several daily life functional tasks, not only isolated activities, using a single device (a smartphone) in a single test.

Regarding the performance of mobility test with biomechanical devices in stroke subjects, Bonnyaud et al. [87] used an optoelectronic motion capture system and David et al. [88] used a pair of insoles. High-precision laboratory systems are complex and expensive [89]. Thus, new motion analysis devices are being developed that are smaller and lighter with more data storage space and less time-consuming, and are found to be an alternative to measure patterns of movement in clinical settings [90]. The latest generation of smartphones often incorporates micro-electromechanical inertial systems with accelerometers and gyroscopes, endowing them with an enormous potential for monitoring the parameters of human movement [56, 89]. In addition, the large onboard memory capacity and wireless connectivity for data transfer make modern smartphones ideal candidates for remote health monitoring [56]. Current evidence demonstrates that both classic clinical tests and instrumental measurements are required when assessing balance after stroke, leading to development of a better individualized treatment program [5]. Taking into account our results, the use of both types of tools would also be helpful in analyzing motor impairment during mobility tasks after a stroke. Future research is needed in this aspect. A standardized mobility test, performed using with a smartphone, offers an accessible alternative that increases the value of functional mobility tests by identifying kinematic variables. This test can be used in clinical, community, and home settings [91].

Our results showed that stroke participants also had lower cranio-caudal ranges. A lower cranio-caudal range is associated with greater energy expenditure because of greater mechanical work performed at the lower limb joints [70 ] and may relate to inefficient gait. Other authors have also found lower COM elevation in stroke participants than in controls during gait when supporting their weight with the affected limb. These authors related such alterations to spasticity and muscle weakness, which are frequent after stroke [82, 85]. A marked increase of the paretic calf muscle tone was observed in 19.45% of our stroke population.

The medial-lateral range significantly correlated with all the clinical tests studied, and it discriminated between more and less impaired stroke participants. Current evidence illustrates that the COM is a kinematic variable that is useful in detecting gait alterations after stroke [85]. Furthermore, COM parameters in the medial-lateral direction are good indicators of dynamic balance, and frontal plane imbalance has been described as a major consequence of stroke [82]. In addition, both the range of over acceleration and the estimated mean power generated when turning around and sitting on the chair were significantly lower in the stroke participants compared to healthy controls. These results can be explained by the fact that ambulatory people with chronic stroke have a marked loss of strength in most major muscle groups of both lower limbs compared to age-matched controls [92]. A negative correlation was found in the turn-to-sit power with the TUG, which is interesting because the TUG seems to be a relevant locomotor task to analyze fall risk, especially in the turning phase [93].

Finally, coinciding with previous literature [94], the reaction time was longer in the stroke participants. It is likely that this difference is not only indicative of an increase in cognitive processing requirements for postural control after stroke, but also occurs instead as a consequence of brain injury [73]. Indeed, the stroke participants showed a worse cognitive status than their counterparts. Walking itself requires cognitive skills, such as looking around, lifting heavy objects, and engaging in social activities. Stroke survivors may experience a greater decline in performance when performing cognitive and motor tasks simultaneously, such as more severe gait deficits and postural instability while walking. This may increase their susceptibility to falls [95]. In this sense, the standardized mobility measure with the Android device allowed for evaluating reaction time in a simple way.

The measurement procedure followed in this study is that established by the Fallskip® system [48], which was originally developed for assessing the functional status of older adults. Previous studies on balance and gait assessment in this population have mainly focused on specific functional assessments, utilizing multiple sensors attached to an external device. However, the Fallskip® system was designed to use simple instrumentation. It consists of a single inertial sensor in a smartphone that can manage the entire process of recording biomechanical variables of clinical interest. With this premise, and based on the literature about assessing balance and gait with portable sensors in older adults [48], it was decided to instrument at the trunk level during its development process. This was because it had been the most commonly instrumented body segment when using a single sensor to monitor an older adult’s activity. Therefore, this system analyzes the kinematics of this single point located near the center of gravity, which is positioned at the level of the S2 sacral vertebra [96]. Due to the challenge of instrumenting this anatomical region with a sensor and the nature of the mobility test, which includes sitting in a chair, the authors of this methodology opted to use the lumbar area at the L4-L5 level to obtain kinematic data from this point. This location enabled the adjustment of a belt around the iliac crests. Likewise, research has demonstrated that the kinematics of the lower lumbar region provide insight into energy expenditure while walking [97]. Furthermore, this sensor location has been previously used in research on the balance and gait of people who have suffered from a stroke. In a review of the use of inertial sensors in assessing balance in neurological diseases [98], 66.6% of the included studies focused on stroke used a single inertial sensor located in the lumbosacral area. Similarly, when smartphones were used for this purpose in stroke survivors, the lumbo-sacral region has also been used [20, 29, 30]. Therefore, the sensor location used in this study is consistent with previous studies on the assessment of walking ability and stability in patients with stroke performed with a single wearable device and the study’ s results have shown the validity of the Fallskip® measurement system in chronic stroke. However, combining multiple data recording devices (e.g. having more than one IMU [99, 100]) may improve the precision of the kinematic variables measured by a smartphone. Nonetheless, this approach could negatively impact the usability and speed of assessments, which are crucial in the clinical context. In addition, it is important to note that current validation studies do not support the use of inertial technology as a substitute for traditional human movement analysis techniques, such as photogrammetry. Therefore, the Fallskip® system, which uses a single sensor on a smartphone, may be valuable in clinical settings for assessing kinematic data during the performance of functional tasks of individuals with stroke. This includes their ability to perform activities of daily living, balance and walking. Hence, clinicians can use this quick, portable and easy-to-use assessment system to monitor their patient’s kinematic parameters during the performance of functional tasks and track its progression.

This study has some limitations that must be pointed out. A purposive sampling was used for the recruitment of the volunteers instead of simple randomization. In addition, generalization of the present results should be limited to the individuals with chronic stroke who could walk at least 10 m with or without a walking aid, which is also a requirement to perform the TUG. It would have been desirable to include a cognitive measure as an inclusion criterion. However, as this would limit the inclusion of subjects with expressive aphasia and right hemiparesis, this requirement was ultimately not considered in order to have a more representative sample of the chronic stroke population. Furthermore, our analyses proved the validity of using a smartphone inertial sensor for assessing kinematic data during the execution of a standardized mobility test in of stroke patients. In this sense, future research about the test reliability in the stroke population is needed, including the precision with which the smartphone is located on the subject. In addition, future research could investigate the influence of different sequelae of people with stroke on their kinematic data during the performance of mobility tasks (i.e., capability of the upper limb, trunk control, cognitive impairment, etc.). On the other hand, although we have used the results of the MAS only as a descriptive variable of muscle spasticity of the paretic calf, caution is required when stating that the MAS is a measure of spasticity. Evidence suggests that resistance to passive movement is not an exclusive measure of spasticity and will vary according to the level of activity in the alpha motor neuron of agonist and antagonist muscles, the viscoelastic properties of soft tissues and joints [101]. Finally, since fewer female patients participated in this study, gender influence could not be investigated.

## Conclusions

This study reports the validity of an Android device in assessing kinematic data in people with chronic stroke and hemiparesis when conducting a standardized mobility test. The inclusion of an easy-to-use inertial sensor embedded in an Android device when performing essential functional mobility tasks facilitated the identification of kinematic differences between people with chronic stroke and their healthy counterparts. Adding the Android device to a standardized mobility test provided insight into the performance in the kinematic parameters of postural control, gait, turning and sitting down, and getting up without spending much more time in the clinical evaluation. All these are relevant and required tasks in the activities of daily living [87]. Assessing all these functional activities in a single test, using only one wearable sensor easily attached to the lower back, implies a qualitative leap in the clinical assessment of kinematic parameters related to functional status in people with chronic stroke. Adding kinematic variables beyond the time required to perform a clinical functional mobility test might be useful for better characterizing functional task biomechanical patterns and better planning intervention approaches.

### Electronic supplementary material

Below is the link to the electronic supplementary material.

Supplementary Material 1

## Data Availability

The datasets used and/or analyzed during the current study are available from the corresponding author on reasonable request.

## Abbreviations

10MWT: 10-meter walk test
5xSTS: Five times sit-to-stand test
Acc: aceleration
APDisp: Anterior-posterior displacement
BMI: Body mass index
CCrange: Cranio-caudal range
COM: Center of mass
COP: Center of pressure
FACHS: Functional ambulation classification of the Hospital of Sagunto
Gyro: gyroscope
ICC: Intraclass correlation coefficient
IMU: inertial measurement unit
IQR: Interquartil range
MAS: Modified Ashworth scale
md: Median
MLDisp: Medial-lateral displacement
MLrange: Medial-lateral range
mn: Mean
mRS: Modified Rankin scale
PStand: Sit-to-stand power
PTurnSit: Turn-to-sit power
RangeJerkSit: Turn-to-sit jerk
RangeJerkStand: Sit-to-stand jerk
SD: standard deviation
TUG: Timed Up and Go test
