# Supplementary material for: Validity of an android device for assessing mobility in people with chronic stroke and hemiparesis: a cross-sectional study
Source: J Neuroeng Rehabil. 2024 Apr 15;21:54. doi: 10.1186/s12984-024-01346-5 (PMC11017601; doi:10.1186/s12984-024-01346-5)
Supplement: Supplementary file 1 — Supplementary Material 1 [file 12984_2024_1346_MOESM1_ESM.docx]

## **CALCULATION OF THE KINEMATIC VARIABLES**

**Postural control variables**

Medial-lateral displacement (MLDisp) and anterior-posterior displacement (APDisp) are calculated by estimating the inclination of the trunk. By considering that the IMU is firmly attached to a position close to the subject's center of gravity, the angular oscillations in the anterior-posterior direction ($\theta_{z}$) and in the medial-lateral direction ($\theta_{x}$) can be measured. The orientations are obtained using Favre's algorithm [1].

To calculate displacements from angular oscillations (1)(2), the IMU's position relative to the ground is used. This information is obtained from the user's anthropometric data, which is previously introduced in the application.

(1) $ML=h\cdot sin(\theta_{z})$

(2) $AP=h\cdot sin(\theta_{x})$

This is a simplified version that was validated using the Kinescan/IBV photogrammetry system (*Instituto de Biomecánica de Valencia*, Valencia, Spain) (Fig 1) [2]. The quadratic errors are less than 5% in the medial-lateral direction and less than 12% in the anterior-posterior direction.

**Fig. 1.** Validation of the center of gravity displacement with the inertial (blue line) and photogrammetry (green line) measurements [2].

Roa: Test of Romberg with eyes open.

**Assessment of gait variables**

As walking is an approximately cyclical activity, the acceleration in the frequency domain (with the gravity component subtracted) is integrated to determine the cranio-caudal (CCrange) and medial-lateral (MLrange) displacements. To achieve this, the acceleration's frequency response is obtained by using the Fourier transform. Then, a double integration in the frequency domain is performed and the displacement signal is calculated by using the inverse Fourier transform (3).

(3) $x\left( \omega\right)=\iint a\left( \omega\right)d\omega=\frac{1}{\omega^{2}}a(\omega)$

The calculated position errors have been less than 5% using this method.

**Assessment of turn-to-sit-to-stand variables**

The mean power was calculated from the segmentation times and the vertical displacement of the center of gravity [2]. Vertical displacement was calculated as the difference between the height of the center of gravity and the height of the chair (45 cm). The height of the center of gravity has been estimated as 55% of the subject's height [3]. Then, power was calculated as in (4) where $P$ is power, $W$ the weight, $\Delta h$ the difference in heights, and $t$ the time.

(4) $P=\frac{W\cdot\Delta h}{t}$

The jerk was calculated by numerically deriving the accelerometer signal in the vertical direction.

**References**

1. Favre J, Jolles BM, Siegrist O, Aminian K. Quaternion-based fusion of gyroscopes and accelerometers to improve 3D angle measurement. Electron Lett. 2006;42:612–4.

2. Pedrero-Sánchez JF. Desarrollo de procedimientos de valoración funcional mediante sensores portables [PhD Thesis]. [Valencia]: Universitat Politècnica de València; 2023.

3. Contini R, Drillis RJ, Bluestein M. Determination of Body Segment Parameters. Hum Factors J Hum Factors Ergon Soc. 1963;5:493–504.
